# SARS-CoV-2 Variants Identification: Overview of Molecular Existing Methods

**DOI:** 10.3390/pathogens11091058

**Published:** 2022-09-17

**Authors:** Giulia Berno, Lavinia Fabeni, Giulia Matusali, Cesare Ernesto Maria Gruber, Martina Rueca, Emanuela Giombini, Anna Rosa Garbuglia

**Affiliations:** Laboratory of Virology, National Institute for Infectious Diseases “Lazzaro Spallanzani” (IRCCS), 00149 Rome, Italy

**Keywords:** SARS-CoV-2, COVID-19, variants characterization, surveillance, sequencing, rapid methods, SARS-CoV-2 pandemic

## Abstract

Since the beginning of COVID-19 pandemic the Real Time sharing of genome sequences of circulating virus supported the diagnostics and surveillance of SARS-CoV-2 and its transmission dynamics. SARS-CoV-2 straightaway showed its tendency to mutate and adapt to the host, culminating in the emergence of variants; so it immediately became of crucial importance to be able to detect them quickly but also to be able to monitor in depth the changes on the whole genome to early identify the new possibly emerging variants. In this scenario, this manuscript aims to provide an overview of the existing methods for the identification of SARS-CoV-2 variants (from rapid method based on identification of one or more specific mutations to Whole Genome sequencing approach-WGS), taking into account limitations, advantages and applications of them in the field of diagnosis and surveillance of SARS-CoV-2.

## 1. Introduction

The new coronavirus SARS-CoV-2 (Severe Acute Respiratory Syndrome Coronavirus-2) is the causative agent of Coronavirus Disease-2019 (COVID-19) [1] and has been responsible for more than 596 million cases worldwide [2].

SARS-CoV-2 has a single-strand positive-sense RNA genome of about 30 kb. Rapid sharing of the first viral genome sequences of SARS-CoV-2 showed that the virus is a member of *Coronaviridae* family, subfamily *Orthocoronavirinae*, genus Betacoronavirus, subgenus Sarbecovirus2 and is closely related to SARS-CoV with which it shares 79% genome sequence identity [3,4]. RNA viruses, such as SARS-CoV-2 have high mutation rates, and their genomes accumulate mutations at a much faster rate than larger organisms [5,6,7]. 

During its rapid spread, several SARS-CoV-2 lineages have emerged with frequent transmission passages. The appearance of these hyper mutated viruses resulted in the change in the infectious characteristics of the pathogen with the increase in transmission capacities [8,9]. In a recent study on the molecular evolutionary characteristics of SARS-CoV-2 in the United States, Wang et al. showed that the nucleotide mutation rate of the whole genome was 6.677 × 10^−4^ substitution per site per year, and the nucleotide mutation rate of the S gene was 8.066 × 10^−4^ substitution per site per year, which was at a medium level compared with other RNA viruses [10]. However, the rate of evolution of the virus gradually decreases over time, considering previous studies about SARS-CoV-2 mutation evolutionary rate [11,12,13,14,15] probably due to its proofreading mechanism, that operates during replication, which can correct some errors that may occur during the copying process, limits the mutation rate of SARS-CoV-2 [16,17].

The SARS-CoV-2 pandemic is the first for which the spread of a virus has been globally tracked in near Real Time using phylogeny of viral genome sequences [11,18]. On 10 January 2020, the first whole-genome sequences of SARS-CoV-2 were made available on GISAID, a database that was already known for its extreme importance for sharing influenza virus sequences from the entire world [12]. 

Since then, around 10 million sequences were deposited online in GISAID that has become the world’s largest repository for SARS-CoV-2 sequences [19]. In particular, Europe shared over 5 million sequences of which around 115,000 from Italy, GISAID facilitates genomic epidemiology and Real Time surveillance to monitor the emergence of new COVID-19 viral strains across the planet [20,21,22,23,24,25,26]. In Italy, to support genomic surveillance of circulating viral variants at regional and national level the IRIDA-ARIES (Integrated Rapid Infectious Disease Analysis - Advanced Research Infrastructure for Experimentation in GenomicS) platform for the collection, analysis and sharing of genomic data of SARS-CoV-2 was created. The structure of the database is divided into regional projects that constitute regional data repositories. The platform accepts genomic sequencing data and sequencing data of the gene coding the Spike protein; performs automatic analysis, returns the results to the user and at the same time returns them to a national archive, equipped with an automatic alert system that is activated when identifying variants of interest to public health or new variants [27]. The analyses of full genomes available on the databases allow to identify the most variable regions and those more conserved, a fundamental information for setting up molecular test. WHO declared that genomic sequencing could help to guide the public health response to a pandemic in near Real Time [28]. 

The sharing of the viral full genome enabled global responses to the pandemic including paramount actions like the development of SARS-CoV-2 diagnostic tests and COVID-19 vaccines and therapeutics [29,30,31,32,33,34]. Indeed, the availability of the genomic sequence of the virus has allowed the development of many laboratory protocols, such as specific SARS-CoV-2 whole-genome amplicon based sequencing protocols and molecular test for rapid SARS-CoV-2 detection. 

SARS-CoV-2 genome sequencing activities are used for the detection of failed vaccinations or re-infections, epidemiological surveillance, identification of new variants, contact tracing and outbreak investigation [28,35,36,37,38,39,40,41,42,43,44,45,46,47,48]. Moreover, the characterization of SARS-CoV-2 genome may guide some therapeutic choices, i.e., for patients who are candidates to the treatment with monoclonal antibodies [49]. The identification of the variant for assistance purposes has led to a request for faster sequencing execution times. 

To respond to this clinical need and rapidly identify SARS-CoV-2 variants, numerous tests which identify the major mutations associated with most variants can be used. 

In this review we summarize the different laboratory approach available for the sequencing and rapid identification of SARS-CoV-2 variants in clinical samples.

## 2. Variants Description 

Since the SARS-CoV-2 virus has been spreading globally, variants have emerged and have been identified in many countries worldwide [50]. 

The World Health Organization (WHO) identified a Working Group of expert that in collaboration with national authorities, institutions, and researchers have been monitoring the evolving situation of the virus. The emergence of variants made it necessary to introduce a nomenclature to distinguish them. Originally, the scientist identified these emerging variants using genetic lineages determined by programs based on phylogenetic framework such as GISAID [51], Nextstrain [52] or Pango [53]. However, often non-scientific audiences associated the variant names with the countries where they were first identified, therefore to avoid episodes of stigmatization towards those countries, the key variants have been associated with letters of the Greek alphabet. The World Health Organization (WHO) working group had the task of define the naming scheme and monitoring the variants evolution. They distinguished the mutated genomes in variants of interest (VOI), which are kept under observation but do not cause concern at the moment, and variants of concern (VOC) for their established increased transmissibility, severity or ability to evade the immune system [54,55,56]. Over time, some variants were de-escalated by European Center for Disease Control and Prevention (ECDC) or became variants being monitored (VBM) by Center for Disease Control and Prevention (CDC), due to the no longer circulation, its low impact on the overall epidemiological situation, or the scientific evidence that the variant is not associated with any concerning property. 

Europe attended to 4 different waves due to the spread of 3 principal VOC, each of which have replaced the precedent variant. 

In late 2020, a variant, initially detected in the United Kingdom, became the most prevalent in Europe, in December 2020 it was awarded the status of VOC by ECDC, and based on the newly nomenclature proposed by the WHO, was named Alpha variant (B.1.1.7) [57]. The prevalence of this variant in the United States, California and Florida reached between 30% and 47% [58].

Later on, due to the drastically reduction of its circulation in Europe, Alpha variant was re-classified as de-escalated variant. The main reasons were the incredible spread of Delta variant, and the evidence that currently available vaccines were highly effective in preventing the infection from Alpha [59].

On May 2021 Delta variant (Pango lineage B.1.617.2) from VOI became a VOC until 7 June 2022 [60], and, months later, a new variant, Omicron (Pango lineage B.1.1.529), established and was promptly classified as VOC in November 2021. BA.2, BA.4 and BA.5 Omicron sub-lineages are currently (to August 2022) the only VOC considered by WHO. From November 2021 to December 2021, Delta and Omicron variants were simultaneously circulating, January 2022, Omicron became the dominant variant and sub-lineages have been identified. 

**Figure 1:**
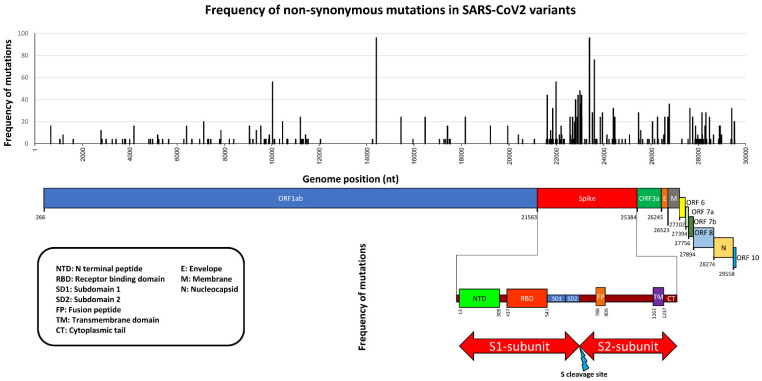
Non-synonymous mutations of SARS-CoV-2 variants. Mutation frequencies and nucleotide positions are reported together with corresponding gene locations. Graphic representation of Spike gene principal domains is also represented.

The Omicron variant has more than 50 mutations, including 26–32 amino acid substitutions, deletions, and insertions [61]. A lineage is a set of closely related variations that share a common ancestor, and these may then branch off into sub-lineages, as seems to be occurring with Omicron. The Omicron variant is considered to have divided into six sub-lineages – BA.1, BA.1.1, BA.2, and BA.3, BA.4 and BA.5, which will continue to change in the future [62,63,64,65,66,67,68]. The four Omicron lineages were first identified in South Africa and Botswana, and has been detected in at least 185 nations and European countries [57]. See Figure 1 and Table 1 for variants specific mutations [69].

Based on available data of transmission, severity, reinfection, diagnostics, therapeutics and impacts of vaccines, BA.2 sub-lineage should be considered a variant of concern and remain classified as Omicron. The Omicron VOC is currently the dominant variant circulating globally, accounting for nearly all sequences reported to GISAID. BA.2 differs from BA.1 in its genetic sequence, including some amino acid differences in the spike protein and other proteins. Studies have shown that BA.2 has a growth advantage over BA.1. Studies are ongoing to understand the reasons for this growth advantage, but initial data suggest that BA.2 appears inherently more transmissible than BA.1, which currently remains the most common Omicron sub-lineage reported [70,71,72,73]. This difference in transmissibility appears to be much smaller than, for example, the difference between BA.1 and Delta. Further, although BA.2 sequences are increasing in proportion relative to other Omicron sub-lineages (BA.1 and BA.1.1), there is still a reported decline in overall cases globally [70].

As of 12 May 2022, ECDC has reclassified Omicron sub-lineages BA.4 and BA.5 from variants of interest to VOC. BA.4 and BA.5 were reported from Gauteng in South Africa in April 2022 [57]. It’s possible that they are fueling a new wave of infections in South Africa, which could eventually break out worldwide. Both BA.4/5 are characterized by mutations in the S receptor-binding domain (RBD), the Q493 reversion mutation (also found in the SARS-CoV-2 Wuhan-Hu 1 strain), and L452R and F486V substitutions. Tuekprakhon A. et al., in a recent study, show as the substitutions in the BA.4/5 RBDs, L452R, and F486V are of most concern because of their potential to confer immune invasion. Both the mutations are close to the angiotensin-converting enzyme 2 (ACE2) receptor surface, hence can modulate RBD-ACE2 affinity and the neutralizing capacity of natural or vaccine acquired immunity. While the reversion mutation Q493, which also lies within the ACE2, likely reduces the escape from responses to earlier SARS-CoV-2 strains [74].

ECDC encourages countries to remain vigilant for signals of BA.4 and BA.5 emergence.

Recombinant strains may emerge when multiple variants infect the same person at the same time, interacting during replication by mixing their genetic materials, giving a new combination.

On 9 March 2022 the WHO, in its weekly epidemiological update, has stated the emergence of three recombinant variants of SARS-CoV-2 namely XE (hybrid of BA.1-BA.2), XF (hybrid of Delta-Omicron) and XD (hybrid of Pango lineage, Delta-Omicron) with possibly high rate of transmission that need to be investigated by risk assessment analysis [75,76,77].

The ECDC named variants under monitoring (VUM) the two recombinants XF and XD. XE recombinant was first identified in the UK on 19 January 2022 [74]. It is characterized by Spike and structural protein from BA.2 and the remaining genome by BA.1 [78].

XD recombinant has been found mostly in France, Denmark and Belgium. It was firstly detected in France and is characterized by the S-protein of BA.1 and the remaining genome from Delta variant. XF recombinant was mostly identified in London and is characterized by the Spike and structural proteins from BA.1 but the 5′ part of its genome is from Delta [15, 57].

Variants listed here must be present in at least one outbreak, detected in a community within the EU/EEA, or there must be evidence that there is community transmission of the variant elsewhere in the world [56]. 

Transmissibility and disease outcome of these recombinants are to date under investigation [79,80].

Sequencing has already proved essential in identifying SARS-CoV-2 as the causative agent of COVID-19 and in investigating its global spread. Virus genome sequences have been used to investigate outbreak dynamics, including changes in the size of the epidemic over time, spatiotemporal spread and transmission routes. In addition, genomic sequences can help the design of diagnostic assays, drugs and vaccines, and to monitor whether changes in their efficacy may be attributable to changes in the virus genome [28].

## 3. Detection and Characterization of SARS-CoV-2 Variants by Sequencing

Whole Genome Sequencing (WGS) of SARS-CoV-2 by Next Generation Sequencing (NGS) technology is the gold standard in the monitoring and the identification of the new variants [28]. Guidance on sampling and sequencing strategy of SARS-CoV-2 can be found in ECDC and WHO technical guidance on sequencing [28,81].

In particular, NGS gives the opportunity to sequence all SARS-CoV-2 genes, including those coding for non-structural proteins and for the intra genes regions. The analysis of the available shared genomes allowed to identify immediately the most conserved regions of the virus, a useful information for diagnostic method design. Moreover, the sequencing of the whole genome, obtained using NGS, is used to carry out phylogeny studies that deliver a more accurate results in comparison to that obtained through the use of partial genes regions. These kinds of analyses are crucial to monitor the mutational trends among the circulating strains in order to identify the possible emergence of new variants [82]. 

In fact, SARS-CoV-2 WGS approach can also sequence new variants that could be different from previous strains, in contrast to the classic molecular methods that are specific sequence-based.

Furthermore, NGS is the most suitable approach to characterize minority variants (Variants containing mutations at <50% frequency) that may be useful for monitoring escape variants in immunosuppressed patients or in patients treated with monoclonal antibody [80,81,82,83,84,85,86].

**Figure 2:**
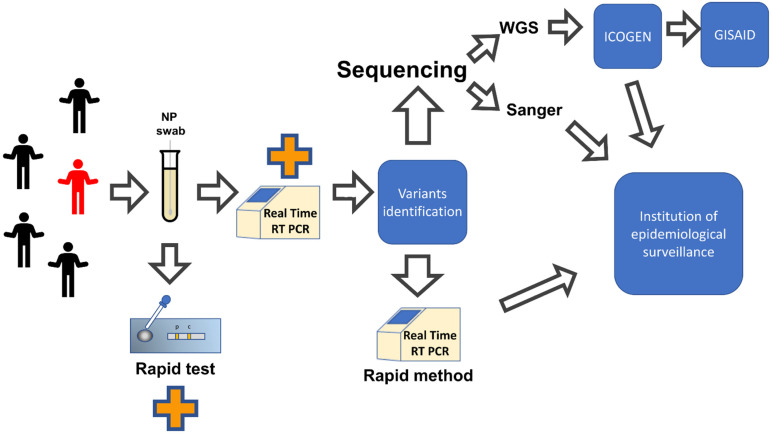
Pipeline of sample analysis in epidemiological surveillance. The sample of different individuals are analysed with RT-PCR or Rapid Test. Only the sample positive to RT- PCR with adequate viral load (Ct < 33) are further analysed with Sanger, NGS or Real Time RT-PCR. The variant identified results are then send to the surveillance authority.

Two different approaches can be used to sequence the entire genome of SARS-CoV-2: targeted (amplicon based) and non-targeted (metagenomics). At the beginning of the pandemic, the entire genome sequence of the virus that caused the pneumonia in China patients, was obtained using a metagenomic approach [3]. These methods allow to obtain the full genome without previous knowledge of the sequence. Successively, several laboratories, included the Italian Istituto Nazionale per le Malattie Infettive (INMI) “Lazzaro Spallanzani”, used this approach to sequence SARS-CoV-2. In particular, the first two imported Italian cases of COVID-19 (January 2020) were sequenced with the metagenomics approach using the SISPA protocol (Sequence Independent Single Primer Amplification) for the libraries preparations which were then sequenced with the ion torrent method, leading to obtaining 40 million reads per sample [87]. A description of pipeline of samples analysis is reported in Figure 2.

The sharing of the obtained genomic sequences had subsequently allowed designing primers that amplify multiple overlapping amplicons to cover entire genome sequence (targeted approach). This method has several advantages such as being less expensive and allowing the simultaneous processing of multiple samples for each single run, making it more efficient in terms of time and cost savings. Different WGS protocols based on amplicon approach have been developed for different sequencing platforms; especially Ion Torrent, Illumina, and Oxford Nanopore Sequencers.

One of the most used primers set was designed by ARTIC Network in March 2020. The advantage of ARTIC primers is that they are open-source, helping the scientific community to improve the capacity of amplify and sequence the entire genome of SARS-CoV-2. The first version of ARTIC primers was based on Wuhan-01 reference genome sequence (GenBank Accession N: MN908947). To date, considering the emergence of several variants that has occurred over time, the primers have been updated with different versions [88]. ARTIC protocol was used for sequencing with different platforms, in particular for protocols developed for Illumina and Oxford Nanopore sequencing platforms (MinION, PromethION, GridION) and protocols based on the use of these primers are provided by various companies (Illumina, NEB, Eurofins, QIAGEN, IDT). However, although approximately 25% of sequences produced came from Oxford Nanopore Technology (ONT), Illumina remain the most commonly used technologies.

This is due to the different efficiency of the results of ONT and Illumina. In fact, in a study of Tshiabuila D. et al. the sequences obtained from the two technologies were compared and the results showed that the ONT sequences had a lower genome coverage and a higher number of indels respect Illumina sequences [89]. 

In March 2020 a panel named “Ampliseq SARS-CoV-2 Research panel” was designed by Life Technologies for Ion Torrent Sequencing, and to date it has been updated to be adapted to the new circulating variants, in particular the omicron which has a very rich and varied pattern of mutations on the S gene; to date the updated version to use for Ampliseq Ion Torrent protocol is the “SARS-CoV-2 Insight Research Assay Panel” (LifeTechnologies) [90].

Plitnick J. et al. assessed the performance of the SARS-CoV-2 AmpliSeq of Ion Torrent vs MiSeq-based ARTIC on 83 clinical samples, identifying that in 81/83 samples the coverages were similar with the two methods. However, they stressed that Ion Torrent workflow is very easily automated with the Ion Chef and S5 instruments, contrary to Illumina [91]. See Table 2 for an overview of commercial available methods for WGS of SARS-CoV-2 by NGS.

Although NGS technologies offer high-throughput and accurate methods for detection of SARS-CoV-2 variants (VOCs, VOI or VUM), the identification of the variants from raw data is complex and requires specialised bioinformatics pipelines. Many laboratories have developed their automatic programs to quickly analyse the raw data, but often they are not user-friendly. 

Jacot et al. proposed a guideline with quality metrics extracted from a typical SARS-CoV-2 Illumina sequencing pipeline. These parameters should help other laboratories to establish simple internal quality controls for SARS-CoV-2 sequencing to avoid wrong interpretation of low quality or contaminated data [92].

Since the beginning of the pandemic, some tools, that had been developed to similar viruses, were used to analysed raw data and sequence reconstruction. One of these was IRMA (Iterative Refinement Meta-Assembler) a robust and reliable assembly program developed by CDC to analyse highly variable RNA viruses [93]. This tool was also integrated in the Ion Torrent pipeline offered to the users with the NGS instrument, until the society had developed own program. 

Instead, Illumina platform analysis is based on DRAGEN (Dynamic Read Analysis for GENomics) pipeline, a commercial tool that allow an efficient analysis of next generation sequencing data [94].

Finally, nanopore technology include the program Guppy to reconstruct the consensus sequences [95].

In many cases, bioinformatics groups of different countries developed tools to process raw data and provide a rapid variants identification. These tools allow to process data came from different sequencing strategy, as capture or amplicon strategy, products both Illumina and Ion Torrent technology [96,97]. Some of these are available online and include automatic workflow that tries to improve the final results [98] (Table S1).

Also INMI bioinformatic group developed a program easy to use to assembly and compare the SARS-CoV-2 genomes [23] that can analyse both Ion Torrent and Illumina raw data. The efficient of the tool was compared to IRMA and DRAGEN pipeline and was identified that ESCA is the tool which best corrects low-coverage regions.

However, in most of these tools, the clades/lineages are assigned by one of two principal tools: Nextclade or Pangolin. These algorithms were developed by the two principal teams of expert (Nextstrain and Pango) that are monitoring and assigning the clades/lineage variation. [53,98]. 

The epidemiological strategy of Italy includes a centralization of raw data analysis using a national analysis pipeline. INMI participated as a Regional Referral Center laboratory to the identification the parameters used in this tool to assess the quality of the consensus sequence. The quality criteria to obtain consensus sequence are essential for a correct variant assignment. In particular, the National Referral Institute, Italian National Institute of Health (ISS), developed the RECoVERY (Reconstruction of CoronaVirus gEnomes & Rapid analYsis) workflow [99] that uses the criteria identified by the sequencing group to reconstruct the consensus sequence. This workflow is now integrated on the I-Co-Gen platform by the ISS, which represents the most up to date system for the collection and analysis of SARS-CoV-2 genome sequencing data in Italy and allow a uniform data analysis at national level [27].

However, although the automatic programmes allow an easy analysis of raw data, sometimes mutation in the new variants could reduce yield of primers amplification. In these cases, a manual control and bioinformatics experience in virology are crucial for the interpretation of the results, especially for the correct assignment of newly emerging variants.

The time and the resources spent in obtaining WGS can cause a delay in gaining results, so in some settings Sanger sequencing of the S-gene can be timelier and more doable than WGS.

Different laboratories developed protocols to amplify specific regions of the S-gene by Reverse Transcriptase (RT)-PCRs followed by sequencing. These regions must identify the characteristic mutations of different VOCs and should ideally include the receptor-binding-domain (RBD) region, but any region covering enough characteristic mutations to conclude that the virus is a specific variant can be used [82].

Salles et al. described feasible protocol for complete nucleotide sequencing of the S gene by Sanger technique using six sets of primers targeting the S segment and two sets flanking it [100].

## 4. Rapid Methods for Early Detection of SARS-CoV-2 Variants

Several methods have been developed for early detection of SARS-CoV-2 variants (VOCs, VOI or VUM). These methods for diagnostic screening mainly consist of nucleic acid amplification technique-based assays able to generate preliminary results in a few hours.

Many of these methods can also accurately identify the variants, while others will require subsequent verification/confirmation by sequencing [82]. We here show and discuss some of the most widely used in diagnostic routine commercially available and home-made methods for variants screening and identification.

### 4.1. Main Commercially Available Methods for SARS-CoV-2 Variants Identification

One of the commercially available methods for identifying variants of SARS-CoV-2 is “COVID-19 Variant Catcher” developed by Clonit S.r.l. This is a qualitative CE-IVD test, based on Real Time RT-PCR, that allows the identification of the S gene mutations 69–70del, E484K and N501Y. The COVID-19 Variant Catcher kit must be used on extracted RNA from SARS-CoV-2 positive samples that have shown amplification within the 35th Cycle Threshold (Ct) in a previous Real Time RT-PCR-based method. The COVID-19 Variant Catcher kit was developed and validated to be used with the following instruments: Rotor Gene Q MDx from QIAGEN, CFX96 from Biorad and 7500 from LifeTechnologies. (Instructions For Use COVID-19 Variant Catcher, Clonit distribuited by Biomedica). An updated version called “COVID-19 Ultra Variant Catcher” discriminates the L452R, E484K, E484Q and N501Y mutations allowing the identification of a larger number of lineages (see Table 3).

Another rapid method for SARS-CoV-2 variants identifications is the “Simplexa™ SARS-CoV-2 Variants Direct assay” (RUO) by Diasorin Molecular, that can identify the SARS-CoV-2 spike mutations N501Y, E484K, E484Q, and L452R. The advantage of this assay consists on its use on nasopharyngeal and nasal swab specimens without previous RNA extraction. Fluorescent probes specific for each mutation are used to perform a post-amplification melting analysis to identify the presence of mutant or wild-type nucleotides at specific locations. This test requires the LIAISON MDX instrument and his appropriate software for the analysis and interpretation of the results. The Limit of Detection (LoD) was determined to be the lowest detectable concentration of quantitated extracted viral genomic RNA (copies/mL) at which ≥ 95% of all replicates test positive and it turned out to be 500 copies/mL. (Instructions For Use of Simplexa™ COVID-19 Direct, by DiaSorin Molecular). We previously examined 118 positive nasopharyngeal swabs (NPS) first characterized by Sanger sequencing, using Simplexa^®^ SARS-CoV-2 Variants Direct assay, with the aim of evaluating the performance of the assay. Results for 111/118 NPS were in complete agreement with the Sanger sequencing, while the remaining 7 samples were not amplified, due to the low viral load. For the 7 NPS not amplified by the assay, a Real Time RT-PCR on thawed samples showed positive results although with high Ct values (median Ct were > 30.for S and ORF1ab gene) [108].

During the last year and a half, with the aim to identify the circulating variants, the Seegene Inc developed a series of multiplex Real Time RT-PCR based typing tests called “Novaplex SARS-CoV-2 Variants Assay” detecting different panels of spike protein mutations such as: 69–70del, E484K, N501Y (Variants I); L452R, W152C, K417T and K417N (Variants II), L452R, P681R, AND K417N (Variants IV), L452Q, F490S,P681R, and L452R (Variants V), L452Q, F490S, R346K, and D950N (Variants VI), and 69–70del, E484A, N501Y and RdRp (Variants VII). The system provides an automated extraction and Real Time RT-PCR setup followed by an automated interpretation reporting data for each specific mutation probe as a Ct value. These assays must be used with Seegene instruments: STARIet (for the extraction and PCR setup), CFX96Dx (for the Real Time RT PCR) and a specific Seegene Viewer (for the automated interpretation). A recent paper compared the results obtained from the Novaplex Variants I, II, and IV assays with S gene Sanger sequencing resulting in a 100% overall agreement in variants identification when using extracted RNA, while a RNA-extraction free protocol was less sensitive in detecting some mutations especially with Ct values > 30 [109].

The ABL (Advanced Biological Laboratories) “UltraGene Assay SARS-CoV-2 Multi Variants Deletions V1” (CE-IVD), a Real Time RT-PCR test intended to be used for the qualitative detection of SARS-CoV-2 deletions 69/70 (Δ69), Y144 (Δ144) and 242–244 (Δ242) on the Spike (S) gene and the deletion 3675–3677 (Δ3675) on the ORF1ab gene in upper respiratory specimens from patients already diagnosed positive to SARS-CoV-2. This system needs extracted RNA by a compatible extraction method (such as Roche MagNA Pure) and, for the amplification stage, is usable with any qPCR instrument compatible with the FAM, HEX, ROX, Cy5 channels. The LoD is 1150 TCID50/mL for the QuantStudio 5 Real Time RT-PCR instrument and 115 TCID50/mL with the UltraGene qPCR 48 instrument.

The “SARS-CoV-2 Extended ELITe MGB” (ELITech Group) molecular kit is a RUO multi-target designed assay intended for use as a reflex test for the detection and discrimination of the mutations L452R, E484K, E484Q and N501Y of the S gene of SARS-CoV-2. This assay foresees a Real Time RT-PCR performed using the ELITe InGenius instrument (ELITechGroup) and the identification of mutations by analysis of the melting curve. 

One of the first commercially available methods intended for the qualitative detection of nucleic acid from SARS-CoV-2 in upper respiratory specimens was “TaqPath™ COVID-19 CE-IVD RT-PCR Kit” (by ThermoFisher Scientific, Waltham, Massachusetts, Stati Uniti). TaqPath™ COVID-19 is a multiplexed assay that contains three primer/probe sets specific to ORF1ab, N gene and S gene of SARS-CoV-2. This system needs extracted RNA (the minimum recommended elution volume is 50 µL). The TaqPath COVID-19 was developed and validated to be used with the following instruments: QuantStudio 5, QuantStudio 7 Flex, 7500 Fast Dx, 7500 Fast, and 7500 from LifeTechnologies. The LoD study established that the lowest SARS-CoV-2 viral concentration (Genomic Copy Equivalents or GCE) that can be detected by the TaqPath™ was 10 Genomic copy equivalent/reaction (GCE/reaction) for both nasopharyngeal swab and bronchoalveolar lavage specimens. The Ct cut-off value for clinical target was ≤37 (TaqPath™ COVID-19 CE-IVD RT-PCR Kit Instructions For Use). Interestingly, although this test was developed with the diagnostic intent of quickly diagnose COVID-19 caused by SARS-CoV-2 infection, if a sample with the 69–70del S-gene mutation is tested using this kit, it will result in an S-gene dropout in presence of ORF1ab and N gene amplifications (also indicated as a S gene Target Failure or SGTF). Considering the importance of this mutation in identifying some SARS-CoV-2 variants, namely the Alpha and Omicron BA.1, this assay, has been used as a fast screening method. On 26 November 2021, the ECDC released a document (“Implications of the emergence and spread of the SARS-CoV-2 B.1.1.529 variant of concern (Omicron) for the EU/EEA”) [110] in which the SGTF from the Thermo Fisher TaqPath assay was indicated as a good proxy for Omicron identification in the scenario present at the time with the Delta variant dominating and the Omicron (BA.1) rising. 

De Pace at al. evaluated the diagnostic performance of five qualitative Real Time RT-PCR based tests (SARS-CoV-2 Variants II Assay—Allplex-Seegene Inc.; UltraGene Assay SARS-CoV-2 452R & 484K & 484Q Mutations V1.x—Advanced Biological Laboratories; COVID-19 Ultra Variant Catcher—Clonit S.r.l; SARS-CoV-2 Extended ELITe MGB—ELITechGroup; Simplexa SARS-CoV-2 Variants Direct - Diasorin Molecular) as compared with NGS finding that the overall accuracy of these assays ranged from 96.9% to 100% and specificity and sensitivity were 100% and 96–100%, respectively. The authors recommend the use of these assays as second-level tests in the routine workflow of SARS-CoV-2 laboratory diagnostics, as they are accurate, user friendly, low cost, may identify specific mutations in about 2–3 h and, therefore, optimize the surveillance of SARS-CoV-2 variants [111].

Vice versa Alejo-Cancho et al. describe three cases in which a Mu strain containing the mutation K417N was initially misclassified as the Beta variant using a multiplex Real Time RT-PCR (Allplex SARS-CoV-2 Variants Assay -Seegene), in this case the authors recommend to use NGS or other methods for the detection of P681H to distinguish between these two variants [112] (Table 3).

### 4.2. In House Rapid Methods for SARS-CoV-2 Variants Identification

Viral genome sequencing procedures are expensive and time-consuming, so with a view to reducing labor intensive and to quickly screen for the different SARS-CoV-2 circulating variants, many laboratories have developed an in house rapid methods for SARS-CoV-2 variants identification. 

Several studies reported delectable results by using in-house molecular tests for identifying a specific single SARS-CoV-2 variant screening, in the contest of knowledge of variants circulating in a given period. Hamill et al. developed a Real Time RT-PCR for deletions of Δ156–157 in the spike gene that is characteristic of the Delta variant with the purpose, primarily to monitor and identify the Delta variant strains, but data analysis indicated that to increase the identification of both Delta and Omicron variants this newly designed assay need to be used in combination with CDC N1 target [119]. 

Barua et al. developed a reverse transcription fluorescence resonance energy transfer-polymerase chain reaction (RT-FRET-PCR) designed to identify the T478K mutation (present in 99.73% of the Delta variant) that can be used both to diagnose COVID-19 patients and simultaneously identify if they are infected with the Delta variant; but the Delta variant is just one of several SARS-CoV-2 variants, so a continuous monitoring of strains will still be necessary [120].

Many other groups have also developed homemade techniques for the identification of single position mutations in order to quickly identify a single variant circulating on the territory [121] but the major limitation of all this kind of assays is the inability to detect all the other major variants because of each variant is characterized by a multitude of mutations. 

Erster et al. focused their attention on developing a test that was able to discriminate between two variants such as Alpha (B.1.1.7) and Beta (B.1.351) variants. This kind of approach can be useful in a territorial context in which two variants mainly circulate but does not allow the identification of new mutation or to the advent of new variants [122].

Because of tracking SARS-CoV-2 variants through WGS can be time consuming and resource-heavy, some laboratories describe an in-house validation of an allele-specific polymerase chain reaction (ASP) variant assay to detect SARS-CoV-2 VOC’s. For example, Brito-Mutunayagam et al. described an ASP based three mutation targets: E484 K, L452R and P681R (based on the circulating variant epidemiology at the time) [123]. Despite the considerable advantage in terms of time, this type of methodology can help to identify a variant only in certain historical periods, when you already know what circulates in a specific area and when you want to discriminate between specific variants.

Other groups describe multiplex Real Time RT-PCR homemade methods capable to detect 9 mutations with specific primers and probes, these PCR typing strategy allowed the detection of the major variants and also provided an open-source PCR assay which could rapidly be deployed in laboratories around the world [124].

Given that genotyping approaches are rapid methods for monitoring SARS-CoV-2 variants but require continuous adaptation, fragment analysis may represent an approach for improved SARS-CoV-2 variant detection. Clark et al. described a multiplex fragment analysis approach using PCR targeting variants by size and fluorescent color. Eight SARS-CoV-2 mutational hot spots in VOCs were targeted. This kind of method could classify a variant with similar accuracy as sequencing without frequent target modification [125].

Moreover, given the possibility that Emerging variants pose the risk for target dropout and false-negative results secondary to primer/probe binding site (PBS) mismatches. Hernandez et al. described a method that combine RT-PCR and matrix-assisted laser desorption/ionization time-of-flight mass-spectrometry to probe for five targets across N and ORF1ab genes, which provides a robust platform to accommodate PBS mismatches in divergent viruses [126].

Another important goal could be to develop a single platform with both diagnostic and surveillance capabilities for comprehensive SARS-CoV-2 Spike gene mutations. This is what was made by Welch et al. that selected up to 26 mutations to distinguish between or detect mutations shared among the Alpha, Beta, Gamma, Delta, Epsilon, and Omicron variant lineages in a cost-effective virus and variant detection platform, which combines CRISPR-based diagnostics and microfluidics with a streamlined workflow for clinical use [127] (Table 4).

**Table 4:** Characteristics of in house rapid methods for SARS-CoV-2 variants identification.

Method	Amino Acid Mutation Detected	Variants Identified	Ref
Real-Time RT-PCR	Δ156–157	B.1.617.2	[117]
RT-FRET-PCR	T478K	B.1.617.2	[118]
One-step RT-qPCR	N501Y	B.1.1.7 B.1.351 P.1 BA.1/2/4/5 (no discrimination)	[119]
Multiplex RT-qPCR	D3L and Δ242–244	B.1.1.7 or B.1.351	[120]
Allele-specific polymerase chain reaction (ASP)	E484 K, L452R and P681R	B.1.1.7, B.1.351, B.1.617.2	[121]
Multiplex RT-qPCR	Δ69–70, K417T, K417N, L452R, E484K, E484Q, N501Y, P681H, and P681R	B.1.1.7, B.1.351, P.1, B.1.617.2, BA.1	[122]
CoVarScan Multiplex fragment analysis (Fluorescently labeled RT-PCR amplicons analyzed by capillary electrophoresis)	8 defined hotspot regions: 5 recurrently deleted regions (RDRs; S:RDR1, S:RDR2, S: RDR3–4, ORF1A, and ORF8) and 3 SNPs (S:N501Y, S:L452R, and S:E484K)	B.1.1.7, B.1.351, P.1, B.1.617.2, BA.1	[123]
RT-PCR/MALDI-TOF	five targets: N1, N2, N3, ORF1A, ORF1AB.	B.1.1.7 (tested december 2020 to april 2021)	[124]
Multiplex CRISPR-based diagnostics and microfluidics	D614G, Δ69–70, N501Y, A570D, P681H/R, D80A, K417N, K417T, L18F, E484K/A, H655Y, P26S, Δ156–157, T478K, L452R/Q, S477N	B.1.1.7, B.1.351, P.1, B.1.617.2, BA.1	[125]

## 5. Concluding Remarks

The prompt production and sharing of a consistent number of SARS-CoV-2 sequences has allowed an accurate diagnosis of the circulating strains and has helped the development of molecular tests for the detection of viral variants. An efficient molecular surveillance approach is essential for the proper management of COVID-19 pandemic since it facilitates the early initiation of effective strategies to mitigate and contain outbreaks of SARS-CoV-2 variants (Figure 2). In alternative to Sanger and NGS methods, several molecular assays have been set up for variants determination which are based on the detection of specific mutations associated with a certain variant. 

Overall the multiplex real-time qualitative RT-PCR based assays for SARS-CoV-2 variants identification target some of the mutations in the spike protein associated with the most widespread variants –e.g., Alpha, Beta, Gamma, Delta, Kappa, and Omicron- and represent simple, accurate and fast methods, enabling high speed detection of known key viral variants. However, all these assays only identify the presence/absence of specific mutations and do not allow to uniquely classifying all viral strains. Indeed, some mutations are common to different variants (e.g., the presence of N501Y + E484K mutations is common to Beta and Gamma variants), and this may lead to an erroneous identification. Moreover, care must be taken in identifying/classifying the viral variants only on the basis of single specific mutation position. The main limit of the rapid typing methods here reviewed relays in the only identification of known variants and the inability to recognize variants resulting from recombination or newly emerged variants on the territory; as a consequence, a continuous update of the assays with the emergence of novel strains is needed and WGS approach by NGS has to be implemented as it represents the gold standard for variants identification and allows to have a complete and accurate picture of the circulation of SARS-CoV-2. 

## Figures and Tables

**Table 1:** The main mutations in the S gene described in the variants of greatest interest.3. Detection and Characterization of SARS-CoV-2 Variants by Sequencing.

Lineage ->		Alpha	Beta (B.1.351)	Gamma (P.1)	Zeta	Eta	Kappa (B.1.617.1)	Delta (B.1.617.2)	Delta (AY.1)	Delta (AY.2)	Delta (AY.4)	Delta (AY.4.2)	Delta (AY.43)	Lambda	Mu	Omicron	Omicron (BA.2)	Omicron (BA.4/BA.5)
		(B.1.1.7)			(P.2)	B.1.525)								(C.37)	(B.1.621)	(BA.1)		
Gene	Spike Regions/Domains	Mutations																
Spike (1273 aa)	NTD (aa13-304)		L18F	L18F														
								T19R	T19R	T19R	T19R	T19R	T19R				T19I	T19I
				T20N														
																	LPP24-26del	LPP24-26del
																	A24S	A24S
				P26S														
						Q52R												
						A67V										A67V		
		HV69-70del				HV69-70del				V70F						HV69-70del		HV69-70del
														G75V				
														T76I				
			D80A															
							T95I				T95I	T95I			T95I	T95I		
				D138Y														
							G142D		G142D	G142D	G142D	G142D	G142D			G142D	G142D	G142D
																VYY143-145del		
															ins144-145VN			
		Y144del				Y144del												
												Y145H						
															Y145N			
							E154K											
								FR157-158del	FR157-158del	FR157-158del	FR157-158del	FR157-158del	FR157-158del					
				R190S														
																N211del		
																L212I		
																	V213G	V213G
																ins215-217EPE		
			D215G															
										A222V		A222V						
			LAL242-244del															
														RSYLTPG246-252del				
			R246I															
									W258L									
	RBD (aa319-541)															G339D	G339D	G339D
																S371L	S371F	S371F
																S373P	S373P	S373P
																S375F	S375F	S375F
																	T376A	T376A
																	D405N	D405N
																	R408S	R408S
			K417N	K417T					K417N	K417N						K417N	K417N	K417N
																N440K	N440K	N440K
																G446S		
							L452R	L452R	L452R	L452R	L452R	L452R	L452R	L452Q				L452R
																S477N	S477N	S477N
								T478K	T478K	T478K	T478K	T478K	T478K			T478K	T478K	T478K
			E484K	E484K	E484K	E484K	E484Q								E484K			
																E484A	E484A	E484A
																		F486V
														F490S				
																Q493R	Q493R	
																G496S		
																Q498R	Q498R	Q498R
		N501Y	N501Y	N501Y											N501Y	N501Y	N501Y	N501Y
																Y505H	Y505H	Y505H
	CTS1/S2 (aa543-1273)															T547K		
		A570D																
		D614G	D614G	D614G	D614G	D614G	D614G	D614G	D614G	D614G	D614G	D614G	D614G	D614G	D614G	D614G	D614G	D614G
				H655Y												H655Y	H655Y	H655Y
						Q677H												
																N679K	N679K	N679K
		P681H					P681R	P681R	P681R	P681R	P681R	P681R	P681R		P681H	P681H	P681H	P681H
			A701V															
		T716I																
																N764K	N764K	N764K
																D796Y	D796Y	D796Y
																N856K		
														T859N				
						F888L												
								D950N	D950N	D950N	D950N	D950N	D950N					
																Q954H	Q954H	Q954H
																N969K	N969K	N969K
																L981F		
		S982A																
				T1027I														
							Q1071H											
		D1118H																
				V1176F	V1176F													

**Table 2:** Commercial WGS Tecnologies comparison.

Tecnology	NGS Platform	Kit for Library Preparation	Methodology	Characteristics and Data Output	Time for Library Prep, Sequencing and Analysis
Illumina	NovaSeq 6000; NextSeq 2000; NextSeq 500/550; NextSeq 550Dx (in RUO mode)	Illumina COVIDSeq Test [101]	Amplicon based	The Illumina COVIDSeq Test leverages a modified version of the validated, publicly available ARTIC multiplex PCR protocol, with 98 amplicons designed to amplify SARS-CoV-2 virus-specific sequences	Library prep: 6–9 h Sequencing:12–44 h depending on flowcell and platform
	All Illumina platforms	AmpliSeq for Illumina SARS-CoV-2 Research Panel [102]	Amplicon based	247 amplicons in 2 pools; >99% coverage of the Coronavirus genome (~30 kb) and covers all potential serotypes	Library prep: <9 h Sequencing:12–44 h depending on flowcell and platform
	MiniSeq; MiSeq, MiSeq; NextSeq 550	Respiratory Virus Oligo Panel [103]	Enrichment	Targets and characterizes ~40 common respiratory viruses, including SARS-CoV-2. hybrid–capture methods allows for highly sensitive detection; near-complete sequence data of targets	Library prep: <9 h. Sequencing: 12–30 h depending on flowcell and platform
	MiniSeq, MiSeq, MiSeq, NextSeq 550	Respiratory Pathogen ID/AMR Enrichment Panel Kit [104]	Enrichment	>280 respiratory pathogens, including SARS-CoV-2; Report full genome coverage of SARS-CoV-2	Library prep: <9 h. Sequencing: 12–30 h depending on flowcell and platform
Ion Torrent	GeneStudio S5/S5 Prime	Ion AmpliSeq SARS-CoV-2 Insight Research Assay [105]	Amplicon based	Two pools with amplicons ranging from 125 bp to 275 bp in length and covers >99% of the SARS-CoV-2 genome, including all serotypes.	Less than 24 h from nucleic acid to data (with overnight Ion Chef System run)
	Genexus Integrated Sequencer (Automated sequencer)	Ion AmpliSeq SARS-CoV-2 Insight Research Assay [105]	Amplicon based	Two pools with amplicons ranging from 125 bp to 275 bp in length and covers >99% of the SARS-CoV-2 genome, including all serotypes.	Library prep plus sequencing and Analysis: <24 h With <30 min hands-on time.
Oxford Nanopore	MinION; GridION; PromethION.	ARTIC protocol [106,107]	Amplicon based	Two pools with amplicons for cover entire genome of SARS-CoV-2	Library prep: 6–9 h Sequencing: it depends on the experiment goal and samples multiplexing

**Table 3:** Characteristics of commercially methods for SARS-CoV-2 variants identification.

Company	SARS-CoV-2 Variants Assay’s Name	Regulatory Approval Status	Including RNA Extraction	Turnaround Time	Limit of Detection or Ct Cutoff Value	Mutation Detected	Interpretation of SARS-CoV-2 Variants Based on Detection of Mutations
ABL Advanced Biological Laboratories	UltraGene Assay SARS-CoV-2 Multi Variants Deletions V1	CE - IVD	No	<1.30 h	1150 TCID50/mL (QuantStudio 5) 115 TCID50/mL (UltraGene qPCR)	69-70del, Y144del, 242-244del, 3675-3677del	69-70del + Y144del + 3675-3677del --> B.1.1.7 242-244del + 3675-3677del --> B.1.351 3675-3677del --> P1 Lineage
Clonit S.r.l.	COVID-19 Variant Catcher COVID-19 Ultra Variant Catcher	CE - IVD	No	≈2 h <1.30 h	SARS-CoV-2 Positive RNA Ct < 35 SARS-CoV-2 Positive RNA Ct < 30	69-70del, E484K, N501Y L452R, E484K, E484Q, N501Y	69-70del + N501Y --> B.1.1.7 E484K + N501Y --> B.1.351/P1 Lineage N501Y --> B.1.1.7 N501Y + E484K --> B.1.351/P.1 L452R --> B.1.617.2/B.1.427/B.1.429/B.1.526/B.1.526.1
Diasorin Molecular	Simplexa™ SARS-CoV-2 Variants Direct assay	RUO	Yes	<2 h	500 copies/mL	N501Y, E484K, E484Q, L452	N501Y + E484K --> B.1.351/P.1 L452R --> B.1.617.2/B.1.427/B.1.429/B.1.526/B.1.526.1 N501Y --> B.1.1.7 E484K -->B.1.525/P2 L452R + E484Q --> B.1.617.1/B.1.617.3 N501Y + E484Q --> B.1.621
ELITech Group	SARS-CoV-2 Variants ELITe MGB^®^ Kit SARS-CoV-2 Extended ELITe MGB^®^ Kit	RUO	No	INA	INA	E484K, N501Y L452R, E484K, E484Q, N501Y	N501Y --> B.1.1.7 N501Y + E484K --> B.1.351/P.1 E484K -->B.1.525/P2 N501Y --> B.1.1.7 N501Y + E484K --> B.1.351/P.1 L452R --> B.1.617.2/B.1.427/B.1.429/B.1.526/B.1.526.1
Life Technologies	TaqPath™ COVID 19 CE IVD RT PCR Kit	CE - IVD	No	<1.30 h	10 GCE/reaction	69-70del	69-70del --> B.1.1.7/B.1.525/BA.1
Seegene	Allplex™ SARS-CoV-2 Variants I Assay	CE - IVD	No (possibility of automated extraction and PCR setup)	INA	INA	69-70del, E484K, N501Y	69-70del + N501Y --> B.1.1.7/B.1.1.529
							E484K + N501Y --> B.1.351/P1/P.3
							69-70del + E484K --> B.1.525
	Allplex™ SARS-CoV-2 Variants II Assay	CE - IVD in progress		INA		L452R, W152C, K417T, K417N	K417N --> B.1.351/B.1.1.529/AY.1/BA.2
							K417T --> P.1
							L452R + W152C --> B.1.429/ B.1.427
							L452R --> B.1.617.1/ B.1.617.2
	Novaplex™ SARS-CoV-2 Variants Assays I	RUO		4 h (starting from extraction)		69-70del, E484K, N501Y	69-70del + N501Y --> B.1.1.7/B.1.529
							E484K + N501Y --> B.1.351/P1/P.3
							69-70del + E484K --> B.1.525
	Novaplex™ SARS-CoV-2 Variants II Assay					L452R, W152C, K417T, K417N	K417N --> B.1.351/B.1.1.529/AY.1/BA.2
							K417T --> P.1
							L452R + W152C --> B.1.429/ B.1.427
							L452R --> B.1.617.1/ B.1.617.2
	Novaplex™ SARS-CoV-2 Variants IV Assay					L452R, P681R, K417N	L452R + P681R --> B.1.617.1/ B.1.617.2
							K417N --> B.1.351/B.1.1.529/BA.2
							L452R + P681R + K417N --> AY.1
							L425R --> B.1.427/B.1.429
	Novaplex™ SARS-CoV-2 Variants V Assay					L452Q, F490S,P681R, L452R	P681R + L452R --> B.1.617.1/B.1.617.2/AY.1
							L425R --> B.1.427/B.1.429
							L452Q+F490S --> C.37
	Novaplex™ SARS-CoV-2 Variants VI Assay					L452Q, F490S, R346K, D950N	D950N --> B.1.617.1/ B.1.617.2/AY.1
							L452Q + F490S --> C.37
							R346K + D950N --> B.1.621
	Novaplex™ SARS-CoV-2 Variants VII Assay					69-70del, E484A, N501Y, RdRp	69-70del + N501Y + RdRp --> B.1.1.7
							N501Y + RdRp --> B.1.351/P.1/P.3
							69-70del + E484A + N501Y + RdRp--> B.1.1.529
							E484A + N501Y + RdRp --> BA.2
							RdRp --> B.1.617.2/AY1/B.1.427/429/P.2/B.1.526/B.1.617.1/C.37/B.1.621
							69-70del + RdRp --> B.1.525

Source: ABL Advanced Biological Laboratories [113] Clonit S.r.l. [114]; Diasorin Molecular [115]; ELITech Group [116]; Life Technologies [117]; Seegene [118]; INA: Information not available.

## Data Availability

Not applicable.

## Supplementary Materials

The following supporting information can be downloaded at: https://www.mdpi.com/article/10.3390/pathogens11091058/s1, Table S1: Summary of analysis tools for SARS-CoV-2 whole genome reconstruction.
