# Supplementary material for: SARS-CoV-2 Variants Identification: Overview of Molecular Existing Methods
Source: Pathogens. 2022 Sep 17;11(9):1058. doi: 10.3390/pathogens11091058 (PMC9504725; doi:10.3390/pathogens11091058)
Supplement: Supplementary file 1 [file pathogens-11-01058-s001.zip › pathogens-1861059-supplementary.pdf]

Table S1. Summary of analysis tools for SARS-CoV-2 whole genome reconstruction.

Program consensus reconstruction	Type of data	Clade/lineage identrification	Note:
**IRMA [96]**	Illumina / Ion Torrent	Pangolin; nextclade	open source program, included in ion suite
**Dragen [97]**	Illumina	Pangolin; nextclade	included in illumina program analysis
**ASPICov [99]; poreCov [98]; ESCA [23]**	Illumina/ Ion Torrent/ Nanopore	Pangolin; nextclade	open sorce program
**RECoVERY [102]**	Illumina/ Ion Torrent	Pangolin; nextclade	Italy analysis program
**EDGE COVID-19 [100]**	illumina/ Nanopore	Pangolin; nextclade	web platform
